# The impact of childhood maltreatment on aggression, criminal risk factors, and treatment trajectories in forensic psychiatric patients

**DOI:** 10.3389/fpsyt.2023.1128020

**Published:** 2023-11-30

**Authors:** Marijtje Koolschijn, Marija Janković, Stefan Bogaerts

**Affiliations:** ^1^Fivoor Forensic Psychiatric Center (FPC) de Kijvelanden, Portugal, Netherlands; ^2^Fivoor Science and Treatment Innovation (FARID), Rotterdam, Netherlands; ^3^Department of Developmental Psychology, Tilburg University, Tilburg, Netherlands

**Keywords:** childhood adversity, childhood maltreatment, ACE, trauma, abuse, neglect, criminality, forensic psychiatric patients

## Abstract

**Introduction:**

Children’s development into healthy well-functioning adults can be negatively affected by adversity. Adverse childhood experiences (ACEs) have been shown to lead to a variety of poor life outcomes, ranging from mental health problems (e.g., anxiety or suicidality) through problematic health behaviors to serious physical diseases and even early death. ACEs can also make people more prone to aggressive behavior, criminality, and recidivism. In this study, we investigated the association between ACEs, specifically childhood maltreatment (CM), and forensically relevant factors; aggression, criminal risk factors, and treatment trajectories, as little is known about these associations in forensic psychiatric patients.

**Methods:**

The study includes data derived from two studies in The Netherlands, of which the first study enrolled 128 patients residing in a Forensic Psychiatric Center (FPC) and the second study included 468 patients who were released unconditionally from FPCs between 2009 and 2013. We expected that more CM would be correlated with higher levels of aggression, higher clinical risk factor scores, and less decrease in clinical risk factor scores over time. To investigate this, we applied correlational analyses and linear growth curve modeling on risk assessment scores and self-report as well as staff report questionnaires on CM and aggression.

**Results:**

Consistent with our first hypothesis, patients with higher CM scores also had higher aggression and risk assessment scores. The effect sizes were small to medium (0.12 to 0.34). Unexpectedly, CM did not influence the course of these treatment trajectories, however, we found that patients with histories of CM had a significantly longer length of stay in a forensic facility than patients without CM (respectively, 10.8 years and 9.3 years on average).

**Discussion:**

This study underlines the importance of carefully examining the history of ACEs and CM in forensic psychiatric patients and considering this in forensic risk assessment and risk guided treatment. More research is needed to draw conclusions about whether and how histories of ACEs should be considered and targeted during treatment trajectories.

## 1 Introduction

Adverse childhood experiences (ACEs) are a major concern in society as they can have a detrimental impact on both individuals and communities. ACEs encompass traumatic events that occur before 18 years of age. They include child maltreatment (CM), and household dysfunction, and can have a lasting negative effect on various life domains (1). For example, the influential “ACE study” by Felitti et al. (1) showed a wide spectrum of mental and physical health problems stemming from ACEs. The study involved nearly 10,000 respondents from the general population and demonstrated that those with higher ACE scores were more susceptible—between four to 12 times—to issues such as alcoholism, drug abuse, depression, and suicide attempts. Additionally, individuals with elevated ACE scores exhibited a heightened risk of developing diseases like autoimmune diseases, cancer, heart and liver diseases, and dying early. Since then, a diversity of studies has replicated these findings across different fields and samples [e.g., (2–6)].

Although ACEs exert a significant negative impact on mental health, the DSM-5 (7) and treatment guidelines do not comprehensively encompass the consequences arising from ACEs. The classification of Post-Traumatic Stress Disorder (PTSD) includes some but not all symptoms associated with pervasive childhood adversity. Various DSM classifications attempt to accurately describe this patient group and their symptoms, ranging from regulatory disorders, attachment disorders, attention deficit and conduct disorders in childhood and adolescence, to personality disorders—especially borderline personality disorder—in adulthood (8, 9). To address this problem, a comprehensive ACE-related classification termed Developmental Trauma Disorder (DTD) was proposed for inclusion in the DSM-5 (10). DTD describes the affect and physiological dysregulation, attention and behavior dysregulation, and self and interpersonal dysregulation that may result from enduring ACEs (11). DTD was not included in the DSM-5 due to ongoing evolution of scientific research (12). Furthermore, the concept of ACEs in the current study is limited to CM: childhood abuse and neglect. Childhood abuse includes emotional abuse (e.g., intimidation or humiliation), physical abuse (e.g., slapping or kicking), and sexual abuse (e.g., unwanted kissing or touching), whereas childhood neglect includes emotional neglect (e.g., disregarding a child’s needs) and physical neglect (e.g., inadequate nutrition or hygiene). These types of childhood abuse and neglect represent ACEs not only within the general population but also within the forensic population, which is the central focus of this study. Many offenders and forensic psychiatric patients reported having been exposed to traumatic or neglectful experiences in childhood and adolescence (13–16). More specifically, individuals who encountered four or more ACEs during their childhood face a 20-fold increase in the probability of being incarcerated than those who did not experience such adversities (14, 17).

The high prevalence of ACEs in offenders and forensic psychiatric patients is not surprising, given the established association between ACEs and the risk of aggression and criminal behavior (18, 19). Aggression, often defined as actions intended to cause harm, pain, or injuries to others (20), has prompted the development of various theoretical models. For instance, the cycle of violence theory supports the notion that early exposure to violence, a component of ACEs, enhances the probability of engaging in violent behavior later in life (21). According to this theory, individuals with ACEs are susceptible to developing behavioral issues, such as uncontrollable impulsive conduct and behavior imitation. As a result, these abused children may grow up to believe that violence is acceptable behavior and hence turn become abusers themselves. Another perspective on the ACEs-aggression link emerges from neurobiological theories suggesting that prolonged childhood and adolescence stress might alter the brain, leading to cognitive impairment and emotional-behavioral dysregulation (22). This, in turn, could heighten the likelihood of adopting an aggressive disposition. Finally, one of the most prominent models explaining human aggression and violence is the General Aggression Model (23). According to this model, personal and situational factors play a role in shaping an individual’s internal state. This, in turn, influences the assessment and decision-making process regarding whether or not to use aggression.

Moreover, aggression is a broad construct, but it can also be further divided into reactive and proactive forms (24). Notably, ACEs are more strongly associated with reactive aggression than proactive aggression (19, 25). Reactive aggression arises as an impulsive emotional response to perceived threats or provocations contrasting with proactive aggression driven by goals and devoid of affect (26). While a cumulative ACE score generally heightens aggression, certain ACE types may also independently contribute to aggressive tendencies. For example, physical abuse has consistently been associated with aggressive behavior in various populations, including the general population, prisoners, and forensic psychiatric patients, while childhood emotional abuse has shown a positive correlation with intimate partner violence [e.g., (27, 28)]. In summary, the association between ACEs and aggression is supported by various theoretical models, as well as empirical studies. As aggression may pose a major obstacle to effective rehabilitation in forensic psychiatry (29, 30), a deeper comprehension of the factors contributing to violent behavior is essential for improved forensic treatment. More research is needed to understand how aggression and its two manifestations are connected to ACEs in general, as well as various ACE subtypes.

In addition to aggression as a behavioral measure, research has illuminated the potential positive correlation between ACEs and specific crime-related risk factors known as dynamic risk factors in criminal behavior (31). Dynamic risk factors, changeable characteristics of offenders and their environments, are expected to increase the likelihood of recidivism. Some examples include impulsivity, addiction, antisocial behavior and hostility (32). Within forensic psychiatry, addressing dynamic criminal risk factors holds pivotal importance, aligning with the goal of reducing recidivism. The Risk Need Responsivity model (RNR) (33) guides this process, outlining how to assess recidivism in forensic patients, suggesting appropriate interventions, and specifying the context for effective interventions. The RNR model (34) states that widely recognized risk factors, such as substance use, antisocial peer relationships, and pro-criminal attitudes underscore the link between ACEs and recidivism, concealing its direct relationship (35). This perspective suggests that ACEs do not directly contribute to recidivism. However, the RNR model also considers the difficulties associated with therapeutically addressing individuals who have committed crimes and experienced traumatic events. Such individuals may have different needs and issues that can influence their responsiveness to treatment [e.g., (36)]. According to the RNR, ACEs are therefore better understood as a responsivity factor in the treatment of offenders.

More recently, an alternative approach emphasizing protective factors has emerged in assessment and treatment, encompassing factors like problem insight, taking responsibility for the crime, social skills and work skills. Attention to protective factors was stimulated by the Good Lives Model (GLM), which is a strength-based approach and represents an extension of the risk-based approach (37, 38). According to the GLM, *inner peace* (i.e., freedom from stress and emotional turmoil), with ACEs conceptually viewed as its opposite end, represents one of 11 intrinsically beneficial needs (i.e., *primary goods*), essential for a fulfilling life (38). Offending, in this framework, represents an unsuccessful endeavor to attain these primary goods. Whether ACEs should be directly integrated into forensic treatment, aligning with the GLM, or only as a responsivity factor, which corresponds to the RNR mode, has triggered debates among researchers and clinicians. Some argue that forensic treatment should be based only on risk factors established by the RNR, while others, including the GLM, advocate for the importance of addressing (complex) trauma using trauma-informed approaches, emphasizing the profound implications for future life and the possible connection to recidivism [e.g., (39)].

That being said, empirical evidence does not consistently align with the assumptions of either the RNR model or the GLM. Research has shown that offenders with a history of ACEs scored higher on criminogenic risk factors in general (16) and impulsivity, addiction, antisocial behavior and hostility in particular (40, 41). As previously stated, ACEs are intricately interwoven into a person’s personality, leaving a lasting impact on physical and neurological functioning. This can eventually lead to psychopathy and cluster B personality disorder in adulthood (32, 42–46), contributing to more criminal behavior and recidivism (47–49). Thus, it is not surprising that many risk assessment tools, such as the Historical-Clinical Risk Management 20, version 3 (HCR-20 ^v3^) (50) or the Historical Clinical and Future—Revised [Historische, Klinische en Toekomstige—Revisie (HKT-R)] (30), consider ACEs as a risk factor for recidivism. However, studies on the association between ACEs or CM and violent recidivism measured with risk assessment tools have shown mixed results. For example, Janković et al. (51) found that ACEs do not significantly influence dynamic HKT-R factors in forensic psychiatric patients with diminished intellectual functioning. Similarly, Spreen et al. (30) found no significant association between CM and general and violent recidivism after 2 and 5 years in a large group of unconditionally released forensic psychiatric patients between 2004 and 2013. Conversely, Heirigs et al. (52) did find a direct link between ACEs and recidivism. In addition, unmet inner peace, considered the opposite of ACEs, was associated with engagement in sexual offending (53) and self-reported drug offending (54). Bouman et al. (55) found that although violent recidivism showed a moderate correlation with unmet needs in general and a strong correlation with poor satisfaction with health at a three-year follow-up, none of these associations remained significant when accounting for risk levels, indicating a preference for risk-based approaches over the GLM. Taken together, these inconsistent results may stem from variations in group compositions, such as differences between prisoners and forensic patients (30, 51). Alternatively, certain studies demonstrating a direct link between ACEs and recidivism may have omitted crucial risk factors in their research (16, 56).

Further research is warranted to ascertain the extent and nature of the association between ACEs and risk factors, as these findings could have clinical implications. The dynamic nature of risk factors complicates the understanding of their interplay with ACEs. Contemporary attention has gravitated toward comprehending alterations in risk and protective factors during forensic psychiatric treatment. Results have shown that risk factors decreased, and protective factors increased during treatment [e.g., (47, 57, 58)]. Empirical evidence from non-forensic psychiatry suggests that patients with ACEs might necessitate prolonged and more intensive treatment for treatment goal attainment (59). This potentially indicates a negative influence of ACEs on treatment progress, which might also be applicable in the forensic context. However, this has not been studied yet.

To our knowledge, the association between ACEs or CM, aggression, risk and protective factors, and treatment progress has not yet been studied in adult forensic psychiatric patients. Therefore, it remains unclear if and how CM impacts this high risk population and the development of (criminal) risk behavior. Better knowledge on this matter could help clinicians improve the delivery and outcomes of forensic psychiatric treatment. The current study, therefore, aims to shed light on these topics. We investigate this using two samples of forensic psychiatric patients residing in or released from forensic clinics in the Netherlands. First, we examine the association between CM and general, reactive and proactive aggression as reported by patients, and general aggression as well as clinical risk and protective factors as reported by staff. Second, we analyze whether CM has an impact on these patients’ forensic treatment trajectories by investigating patients’ progress measured by risk and protective factor scores at three time points from inpatient admission until unconditional release.

Based on the literature summarized above, we expect to find higher levels of general and reactive aggression, but not proactive aggression, in patients with more CM. We also expect that CM is positively associated with clinical risk factors and negatively associated with clinical protective factors at the time of admission. Finally, we hypothesize that these associations change for the better over treatment time but change more slowly in patients with higher levels of CM.

## 2 Methods

### 2.1 Procedure and participants

This study uses two datasets to examine the hypotheses. Both datasets encompass information concerning forensic patients subjected to a “tbs measure” a distinct measure within the Dutch jurisdiction. The term tbs. (“terbeschikkingstelling,” when translated literally, signifies “made available”). Individuals with a tbs. measure are deemed either not fully responsible or only partially responsible for their committed crimes, due to their mental illness. These individuals are mandated to undergo treatment under state authority, specifically for violent crimes warranting a prison sentence of at least 4 years. When held partially responsible, a prison sentence precedes the admission to a tbs. treatment facility known as a Forensic Psychiatric Center (FPC). Tbs treatment of an indeterminate duration, differing from conventional prison sentences which have a defined termination point. Whether patients are able to reintegrate into society depends on whether the treatment succeeds in reducing the risk of recidivism risk as assessed with risk assessment measures (see below). Every 2 years, an evaluation takes place to decide whether the tbs. measure will be extended or terminated (60).

The first dataset, denoted as the “VRAPT dataset” consists of data collected for a randomized controlled trial, investigating the effectiveness of Virtual Reality Aggression Prevention Training (VRAPT) among forensic psychiatric patients. The study included 128 patients from four FPCs, all with a history or current display of aggression. The study was approved by the medical ethical committee of the University Medical Center Groningen (61). We used patient demographic data and self-reported ratings of childhood trauma and aggression. Both baseline data from patients in the waiting list condition and those receiving VRAPT intervention were analyzed. Notably, the current study employed data from all patients included in the VRAPT study, without distinguishing between those who did or did not receive VRAPT. The baseline period refers to a period of 12 weeks before the VRAP intervention. In addition, staff observations on patient aggression during the baseline period were also taken into account.

The second dataset, hereafter referred to as the “HKT dataset,” contains data on patients who have undergone unconditional release following tbs. treatment. The dataset is part of a broader study into treatment evaluation. HKT-R. The dataset contains data from 468 forensic patients who were unconditionally released from an FPC between 2009 and 2013. All data, like patients’ criminal history, risk assessment, psychiatric diagnoses, treatment plans, leave requests, and prolongation of treatment recommendations, were collected retrospectively by trained bachelor and master psychologists, and were anonymized and stored securely. Psychiatric diagnoses were based on the Diagnostic and Statistical Manual of Mental Disorders (4th ed., text rev.; DSM-IV-TR), the prevailing diagnostic framework at the time of the treatment. The study was approved by the ethical review board of Tilburg University, the Ministry of Justice and Security and the scientific research committee of Fivoor (30, 62).

The length of stay in highly secured tbs. facilities spans several years, with a current average duration of eight, and involves tailored therapeutic interventions targeting the specific problem domains of the patients. This includes various modalities, such as psychological, psychiatric, and occupational therapy, as well as training with and practice in all areas of life, like work and finances. Because of this, the type of treatment programs followed during the stay in the FPC differs per patient over both datasets. The central goal of all interventions is aimed at decreasing forensic risk factors, which is used as an outcome measure for treatment success in the current study.

### 2.2 Measures

#### 2.2.1 Childhood maltreatment

In the VRAPT dataset, childhood maltreatment was measured with the Childhood Trauma Questionnaire-Short Form (CTQ-SF) (63). The CTQ-SF is a self-report questionnaire with 25 items scored on a 5-point Likert scale ranging from “never true” (1) to “very often true” (5). Example items are “People in my family said hurtful or insulting things to me” and “I believe I was sexually abused.” The questionnaire produces scores for each patient on five categories of childhood maltreatment up to the age of 18 years: physical, emotional, and sexual abuse; emotional and physical neglect. Outcomes are categorized by severity, ranging from “none to minimal,” to “severe.” The questionnaire was found to be valid and internally consistent in several samples and the validity and reliability (Cronbach’s alpha ≥ 0.63) are adequate [e.g., (64)].

In the HKT-dataset, one of the items from the aforementioned HKT-R was used as a measure of childhood maltreatment. The HKT-R is widely used in Dutch forensic psychiatry and was designated by the Ministry of Justice and Security as a mandatory tool for risk assessment and monitoring. It is comparable to international risk assessment measures like the HCR-20 ^v3^ (50, 65). It measures forensic risk factors across three domains: history (12 items), clinical (14 items) and future (7 items). Its predictive validity in general and violent recidivism was good in a large sample of forensic psychiatric patients discharged unconditionally between 2004 and 2008 from high-security forensic institutions in the Netherlands (62) as well as in forensic psychiatric patients with disabilities in intellectual functioning (51).

The HKT-R historical domain contains one broad item related to childhood maltreatment: victimization before the age of 18 (item “H07”). This was scored at the time of admission to the FPC. In the HKT dataset, in addition to regular use of the HKT-R where H07 is a compound item, different types of victimization have been specified. The eight categories include whether the patient has been a victim of physical, emotional, or sexual abuse; was physically or emotionally neglected, extremely spoiled, prostituted, and witnessed domestic violence in the household of origin. The compound item and the first five of the specified subcategories—the categories corresponding with those of the CTQ-SF—were used in the current study. The compound item is scored on a 5-point scale ranging from 0 to 4, where 0 is “the patient has never been a victim or witness of violence,” 1 is “there has been incidental neglect and/or incidental abuse,” 2 is “there has been chronic neglect (and incidental abuse),” 3 is “there has been chronic abuse (and incidental neglect),” and 4 is “there has been chronic neglect and abuse.” The subcategories are scored dichotomously, either yes or no (1 or 0). The instrument has been found to be valid and consistent (30). The internal consistency of the historical scale is good (Cronbach’s alpha 0.81).

#### 2.2.2 Aggression

Current levels of aggression were measured with data from the VRAPT dataset. Two questionnaires were used, the Reactive Proactive Questionnaire (RPQ) (66) as a self-report measure and the Social Dysfunction and Aggression Scale (SDAS) (67) as a staff-scored measure.

The RPQ measures type and severity of aggression. Respondents are asked 23 questions about types of aggression they display and reasons for doing so. Example questions are “How often did you fight to show who’s the boss?” and “How often did you force someone to give you money or other items?.” Answers are given on a 3-point Likert scale, where 0 is never, 1 is sometimes and 2 is often. The outcome categories are reactive aggression (11 items), proactive aggression (12 items) and a total combined aggression score. The internal consistency of the total scale and reactive and proactive subscales are excellent (Cronbach’s alphas of 0.91, 0.83, and 0.87, respectively) and the questionnaire has been found useful and valid for adults besides its original aim for children (64, 68, 69).

The SDAS, a 9-item version, has staff observe and rate a broad range of aggressive behavior from the patient on a 5-point scale, ranging from 0 = not present to 4 = extremely severe. This includes behaviors like negativity, irritability, verbal aggression, and physical aggression. A weekly peak score and general score in aggression were scored, following the Dutch manual of Bousardt (70). For the current study, the weekly scores averaged per patient were used by calculating a total score and dividing it by the number of weeks the instrument was scored (ranging from 9 to 12 weeks). The reliability of the SDAS is sufficient (Cronbach’s alpha 0.82) and the validity is good (64, 67).

#### 2.2.3 Clinical risk factors

We used the 14 clinical dynamic risk factors derived from the HKT-R clinical domain (“K” items). These are dynamic risk factors that are known to be directly or indirectly related to delinquent behavior and recidivism. The clinical domain total score is used to assess treatment progress and risk of reoffending. As previously mentioned, the items can be categorized into two subscales: a risk subscale and a protective subscale. Both scales include seven items. The risk subscale is a total score of the following clinical HKT-R indicators: psychotic symptoms, addiction, impulsivity, antisocial behavior, hostility, violation of terms and orientation to the criminal milieu. The protective subscale is a reversed total score of the remaining seven clinical HKT-R indicators including problem insight, treatment compliance, taking responsibility for the crime, self-reliance, social skills, coping skills, and work skills. All factors are scored on a 5-point scale ranging from 0 to 4 with higher scores indicating that a particular risk factor is more present in the patient being assessed. The reliability of the clinical scale is good (Cronbach’s alpha 0.83). The HKT-R has several advantages over other national or international risk assessment scales, when it comes to evaluating clinical dynamic risk and protective factors. One notable distinction is that it takes into account a more comprehensive range of clinical factors compared to other scales. This is made possible by the utilization of 14 factors and a detailed five-point scale. Moreover, since it was specifically designed and tailored for the Dutch forensic field, with valuable input from clinicians, the HKT-R stands as a specialized scale for our unique population. Because the HKT-R serves as a mandatory tool for assessing all patients within Forensic Psychiatric Centers in the Netherlands, information derived from the HKT-R is available for the entire cohort of discharged patients.

For the research question on treatment trajectories, the clinical items were used at three time points: admission to the clinic (T1), when patients were given permission for unguided leave (T2), and unconditional discharge at the end of the treatment trajectory, when the tbs. measure is no longer applicable (T3). The mean length of stay was 10.5 years (SD = 4.08), ranging from 2 to 26 years. The time between T1 and T2 was on average 3.9 years (SD = 1.96), and between T2 and T3 6.07 years (SD = 3.83). For the research question on the correlation between childhood adversity and clinical risk and protective factors, only T1 (time of admission to the clinic) was used, as at that time point there was no effect of treatment on the clinical factors yet.

### 2.3 Statistical analysis

Demographics, including the frequency of childhood adversity in both samples, were computed with SPSS version 27 (71).^1^ Before performing the main analyses, data were checked for missing data, outliers, normality, and multicollinearity. Missing data were excluded pairwise for the relevant analyses and outliers were not present in the data. Data were normally distributed (Supplementary Table S1), where absolute values of skewness and kurtosis were not larger than 3 (72). There was no multicollinearity, as measured by Variance Inflation Factors (VIF) and tolerance; a VIF > 4.0 or a tolerance < 0.2 may indicate multicollinearity (73).

To research the association between childhood maltreatment and aggression and clinical risk factors, we used the Pearson correlation coefficient. In the VRAPT sample, the total score and the five CM subcategory scores of the CTQ-SF were correlated with the scores on the RPQ (self-reported inpatient general, reactive, and proactive aggression) as well as the SDAS (mean scores of staff-reported inpatient peak and general inpatient aggression). In the HKT sample, correlation coefficients between childhood victimization (H07 total score) and scores on clinical risk factors (K total score and scores on risk and protective subscales) of the HKT-R were computed using the Pearson correlation coefficient. Point-biserial correlation was used to correlate the five (dichotomous) CM subcategories to the clinical (K) total score and risk and protective subscales. As correlations in one direction were expected, one-tailed correlations were used in both samples. These analyses were also done in SPSS [IBM (71)]. A value of *p* of 0.05 was set as significant. Resulting effect sizes are interpreted as very small (0.01), small (0.2), medium (0.5), large (0.8), and very large (1.2) following Cohen (74) and Sawilowsky (75).

The HKT-R dataset was used to research the treatment trajectory and any influence of childhood maltreatment—as measured by HKT-R H07 childhood victimization—on it. For this, Latent Growth Curve Modeling (LGCM) was applied in the free software environment R (76). LGCM is a statistical technique to model change over time. Changes in dynamic risk and protective factors were researched at the three time points from the moment of admission to the clinic up to the moment of unconditional release. Missing data were treated with full information maximum likelihood and outliers were not present. The minimum sample size for these analyses is at least five observations per estimated parameter, whereby 50 respondents generate sufficient power (77). The analysis was done at scale and subscale levels for the 14 K-items, including a total clinical risk factor score, a protective subscale and a risk subscale. The total score on HKT-R item H07 childhood victimization was dichotomized to create a grouping variable (0 = no ACEs and 1 = one or more ACEs) to allow comparison between groups at each time point. First, an unconditional model was tested to investigate whether a decrease in the clinical scale and risk subscale and an increase in the protective subscale can be found from T1 to T3. Next, a conditional model was used, in which childhood adversity was included as a predictor. This tested whether childhood adversity plays a role as a predictor in the growth model, and the stability of the unconditional model. The model fit was evaluated using the comparative fit index (CFI; values ≥0.90) and standardized root-mean-square residual (SRMR; <0.08) (78). Finally, a post-hoc analysis of variance (ANOVA) was used to investigate the between-group differences (CM vs. no CM) in the clinical scale as well as risk and protective subscales for the three time points. All analyses were controlled for gender.

## 3 Results

### 3.1 Sample characteristics

The VRAPT dataset consists of data on 128 male patients (intention to treat), with a mean age of 39 years and largely of Dutch origin. Their education level is mostly lower education (63.8%). Almost all participants were diagnosed with a personality disorder (most prevalent were antisocial and borderline personality disorder, respectively, 37.5% and 12.5% of the sample), a third with psychotic disorders and a third with developmental disorders. PTSD was diagnosed in 7% of the sample. For a more comprehensive overview of psychiatric diagnoses, see Supplementary Table S5.

Patients’ index offenses were mostly violent, more than 40% committed an attempted or completed homicide. A fourth committed a sexual offense and a fourth a property offense. See Table 1 for an overview (61).

**Table 1:** Sample characteristics.

Characteristic	VRAPT dataset (*n* = 128, 100% males)	HKT-R dataset (*n* = 468, 86.5% males)
	M (SD) or *n* (%)	M (SD) or *n* (%)
Age	38.7 (10.3)	33.2 (9.8) at start tbs.
Institutional length of stay	Not yet discharged	10.5 (4.1) years
Dutch origin	98 (77%)	351 (75%)
**Highest completed education**		
None	10 (8%)	44 (9%)
Special education	17 (13.4)	NA
Lower education	81 (63.8%)	185 (40%)
Secondary education	14 (11%)	191 (41%)
Tertiary education	4 (3%)	16 (3%)
Missing/other	2 (2%)	32 (7%)
**DSM-classification**		
Psychotic disorders	40 (32%)	151 (32%)
Personality disorders	126 (98%)	360 (77%)
Developmental disorders	36 (28%)	44 (9%)
Sexual disorders	13 (10%)	34 (7%)
PTSD	9 (7%)	19 (4%)
Age at index offense	31.3 (9.5)	32.8 (9.6)
**Type of index offense**		
(Attempted) homicide	54 (42%)	216 (46%)
Sexual offense	36 (28%)	90 (19%)
Violent offense	74 (58%)	319 (68%)
Property offense	35 (27%)	49 (10%)
Arson	7 (5%)	59 (13%)

As there is overlap in classifications and offenses, not all percentages add up to 100%. NA, This category was not measured in the HKT-R sample.

The HKT-R dataset consists of data on 468 patients (13.5% female). The mean age at the start of the tbs. was 33 years, ranging from 18 to 79. The mean length of stay in the institution was 10.5 years (SD = 4.08), ranging from 2 to 26 years. The time between admission (T1) and unguided leave (T2) was on average 3.9 years (SD = 1.96), and between unguided leave (T2) and unconditional discharge (T3) 6.07 years (SD = 3.83). Most patients had lower (40%) or intermediate (41%) education. Most of them were single (65.6%), while 17.1% were married, 14.6% divorced and 0.9 widowed. In addition, 37.4% of the patients had children. The most prevalent personality disorders were personality disorder not otherwise specified (34.6%) and cluster B personality disorders (30.6%). Substance use disorders occurred in almost half of the sample, while psychotic disorders occurred in a third of the sample. Developmental disorders and sexual disorders were less prevalent, below 10%. PTSD was diagnosed in 4% of the sample. For a more comprehensive overview of psychiatric diagnoses, see Supplementary Table S6. Many of the index offenses were violent, almost half attempted or completed homicide. A fifth of the patients committed a sexual offense and around 10% committed a property offense or arson. See Table 1 for an overview.

Looking at the prevalence of childhood victimization, the results show that 60.2% of the sample experienced some form of victimization before the age of 18 based on self-reports in the VRAPT study while this percentage is 83.3% of the sample according to the reports made by the professionals in the HKT-R study. For more detail on the prevalence of each ACE category, see Supplementary Tables S2–S4.

### 3.2 Associations between childhood maltreatment and measures of patient reported and staff-reported inpatient aggression in the VRAPT sample

In the VRAPT dataset, we correlated the scores on CM with the self-report measure for inpatient aggression as well as the staff-reported measure for inpatient aggression. The total score on CM was significantly and positively associated with self-reported general aggression, as well as with reactive and proactive aggression, respectively. All subcategories of CM correlated significantly and positively with the total score on self-reported aggression. Physical neglect had the strongest significant association with all three scores on self-reported aggression, followed by emotional and physical abuse. Emotional neglect was positively and significantly associated with self-reported general and proactive, but not reactive, aggression. Sexual abuse was positively and significantly associated only with self-reported general aggression. All significant correlations had a small to medium effect size. No significant associations were found between sexual abuse and self-reported reactive and proactive aggression, and between emotional abuse and reactive aggression. See Table 2 for all results.

**Table 2:** Correlations between CM (CTQ-SF) and aggression (RPQ and SDAS).

Correlation	RPQ total score (*n* = 104)	RPQ reactive aggression (*n* = 104)	RPQ proactive aggression (*n* = 104)	SDAS peak (mean week score) (*n* = 117)	SDAS (mean week score) (*n* = 117)
CTQ-SF total score	**0.254** ^**^	**0.246** ^**^	**0.256** ^**^	0.081	0.110
Emotional abuse	**0.235** ^**^	**0.263** ^**^	**0.218** ^*^	**0.167** ^*^	**0.172** ^*^
Physical abuse	**0.237** ^**^	**0.219** ^*^	**0.252** ^**^	0.141	**0.166** ^*^
Sexual abuse	**0.174** ^*^	0.162	0.157	−0.003	0.024
Emotional neglect	**0.184** ^*^	0.150	**0.191** ^*^	0.013	0.084
Physical neglect	**0.337** ^***^	**0.325** ^***^	**0.320** ^***^	**0.176** ^*^	**0.175** ^*^

CTQ-SF, Child Trauma Questionnaire-Short Form; RPQ, Reactive Proactive Questionnaire; SDAS, Social Dysfunction and Aggression Scale. Significant correlations in bold. ^***^Correlation is significant at the 0.001 level (1-tailed); ^**^Correlation is significant at the 0.01 level (1-tailed); ^*^Correlation is significant at the 0.05 level (1-tailed).

The total score on CM did not correlate significantly with the peak and general average week scores on staff-reported aggression. Looking at the subcategories of CM, however, emotional abuse and physical abuse and neglect did correlate significantly and positively with the SDAS scores. The other categories have no significant correlation. All significant correlations were small. The results are presented in Table 2.

### 3.3 Associations between childhood maltreatment and dynamic clinical risk factors at the time of admission in the HKT sample

In the HKT dataset, we first correlated the total score on H07 (childhood victimization) with the total clinical risk factor score at the time of admission to the FPC (T1). This showed a small, but significant positive correlation (*r* = 0.121, *p = 0.*021). When looking at the two subscales of the clinical HKT-R scale, a similar but significant negative correlation (*r =* −0.120*, p = 0*.020) was found between childhood victimization and the protective subscale. The correlation between childhood victimization and the risk subscale was not significant.

Next, we researched how the five subtypes of childhood victimization correlated with the total clinical risk score and its two subscales. No significant correlations were found. Results are listed in Table 3.

**Table 3:** Correlations between CM (H07—childhood victimization) and clinical risk factor (K-) scores.

Correlation	H07 total (*n* = 468)	Physical abuse (*n* = 468)	Sexual abuse (*n* = 468)	Physical neglect (*n* = 468)	Emotional neglect (*n* = 468)	Emotional abuse (*n* = 468)
Total clinical scale (*n* = 282)	**0.121** ^*^	0.055	0.036	−0.026	0.078	−0.007
Risk subscale (*n* = 286)	0.084	0.032	0.049	0.004	0.068	−0.004
Protective subscale (*n* = 282)	**−0.120** ^*^	0.054	−0.002	−0.028	0.055	−0.021

Significant correlations in bold. ^*^Correlation is significant at the 0.05 level (1-tailed).

### 3.4 Influence of childhood maltreatment on patients’ treatment trajectories in the HKT sample

#### 3.4.1 Unconditional models

We first used LGCM to test an unconditional model for the trajectory of the total clinical risk factor scale from T1 to T3. As this hypothetical model did not fit the data well, we applied an ANOVA repeated measures design to find that the clinical score decreased significantly over the three time points.

Next, we used the total scores for subscales of risk and protective factors derived from the clinical risk factor scale. These subscales have been found and used in previous research [c.f., (37)]. The unconditional model for the risk subscale fitted the data well. The results showed a significant decrease in risk factors during the treatment trajectory from T1 to T3. In contrast, the unconditional model for the protective subscale did not fit the data well and therefore we performed an ANOVA repeated measures design to find that the protective score increased significantly over the three time points. For a more detailed description of these analyses, see Supplementary material.

#### 3.4.2 Conditional models

To test whether the decrease from T1 to T3 of patients’ total clinical risk scale score is dependent on the experience of childhood adversity, we applied a repeated measures ANOVA within- and between-subjects design as the LGCM did not fit the data well (79). Childhood maltreatment—as measured by H07 total (dichotomized)—was included as a between-subjects factor. As we determined while researching the conditional models, the residuals were approximately normally distributed, while the assumption of sphericity was violated, and therefore the Huynh–Feldt correction was applied. The results confirmed that there was a significant effect of time [*F*(1.881, 426.986) = 8.373, *p* < 0.001, η^2^ = 0.036]. However, the interaction between time and childhood maltreatment was not statistically significant [*F*(1.881, 426.986) = 0.023, *p* = 0.971], indicating that CM does not influence the changes in the clinical scale score over time. Gender was not a significant covariate, which means that male and female patients changed on the clinical scale at the same pace. Finally, a post-hoc ANOVA revealed that the two groups did not differ in the total clinical risk scale score at any of the time points (Table 4).

**Table 4:** Differences in risk and protective scores between patients with or without CM.

	Entire sample (*n* = 468)	CM (*n* = 390)	No CM (*n* = 78)	*F* test
		Mean (SD)		
**Total clinical scale**				
T1	20.74 (9.31)	21.18 (9.38)	19.05 (8.94)	*F*(1,287) = 2.146
T2	13.52 (6.87)	13.70 (7.07)	12.79 (5.79)	*F*(1,381) = 0.932
T3	10.40 (7.90)	10.70 (8.13)	9.09 (6.47)	*F*(1,452) = 2.676
**Risk subscale**				
T1	8.43 (5.36)	8.66 (5.33)	7.61 (5.44)	*F*(1,282) = 1.583
T2	5.28 (3.81)	5.44 (3.89)	4.54 (3.28)	*F*(1,391) = 3.165
T3	3.80 (3.93)	3.98 (4.09)	2.93 (2.97)	*F*(1,457) = 4.649^*^
**Protective subscale**				
T1	15.66 (4.91)	15.46 (5.00)	16.56 (4.53)	*F*(1,278) = 2.060
T2	20.34 (4.34)	20.42 (4.41)	19.68(3.96)	*F*(1,198) = 0.710
T3	22.06 (4.87)	21.95 (4.95)	22.59(4.33)	*F*(1,268) = 0.554

T1, admission to clinic; T2, unguided leave; T3, unconditional discharge. ^*^Significant difference at the 0.05 level.

The conditional model was tested for the risk subscale with LGCM, as the unconditional model for this subscale had a good fit to the data. For the risk subscale, the model fitted the data well (CFI = 0.91, SRMR = 0.04), however, childhood adversity was not significantly associated with changes in the risk subscale score. Gender was not a significant covariate either (*b* = −0.44, *p* = 0.41). A post-hoc ANOVA (Table 4) showed that the CM group scored significantly higher on the risk subscale than the non-CM group at T3, whereas there were no significant differences on this subscale at T1 and T2 between the two groups.

Because the unconditional model for the protective subscale did not fit the data well, we again applied a repeated measures ANOVA within- and between-subjects design to test whether there are differences in trajectories of protective score over time between the CM and non-CM groups. The assumption of sphericity was violated, and thus the Huynh–Feldt correction was applied. The results confirmed that there was a significant effect of time [*F*(1.8814, 159.617) = 4.586, *p* < 0.001, η^2^ = 0.050]. However, the interaction between time and childhood adversity was not statistically significant [*F*(1.814, 159.617) = 0.111, *p* = 0.877], indicating that adverse childhood experiences do not influence the changes in the clinical scale score over time. Gender was not a significant covariate.

Table 4 shows a post-hoc analysis of the patients’ risk factor scores during the treatment trajectory. No significant differences were found on the protective subscale between the two groups at any of the time points.

We also calculated the mean institutional length of stay for the CM and no CM group over the full treatment trajectory and between the three time points. There was a significant difference in the length of stay from admission (T1) to unconditional discharge (T3) between the two groups, where the patients with CM stayed significantly longer on average in a forensic facility (10.8 years) than those without (9.4 years). There was no significant difference between the two groups for the period from admission (T1) to unguided leave (T2), in which both groups have an average length of stay of 3.9 years. The period from unguided leave (T2) to unconditional discharge (T3) did show a significant difference, where the average length of stay for the CM group (6.4 years) was significantly longer than the group without CM (4.7 years). The results are listed in Table 5.

**Table 5:** Differences in length of stay between patients with or without CM (HKT-sample).

	Entire sample (*n* = 468)	CM (*n* = 390)	No CM (*n* = 78)	*F* test
		Mean (SD)		
Total average length of stay (T1 to T3)	10.52 (4.08)	10.77 (4.10)	9.36 (3.79)	7.85^**^
Average length of stay from T1 to T2	3.93 (1.96)	3.93 (1.8)	3.89 (2.56)	0.038
Average length of stay from T2 to T3	6.08 (3.83)	6.38 (3.89)	4.68 (3.21)	11.46^***^

T1, admission to clinic; T2, unguided leave; T3, unconditional discharge. CM/no CM as scored on H07—childhood victimization. ^**^Significant difference at the 0.01 level. ^***^Significant difference at the 0.001 level.

## 4 Discussion

### 4.1 Discussion of the results

In this study, we investigated the association of ACEs, specifically childhood maltreatment (CM, with aggression as well as risk and protective factor scores in forensic psychiatric patients). We used two samples of forensic psychiatric patients; 128 patients still residing in FPCs (“VRAPT sample”) and 468 patients unconditionally released from the FPCs (“HKT-R sample”). In addition, we investigated whether CM could influence patient treatment progress from the moment of admission to the clinic up to the moment of unconditional release. Overall, we found that patients with higher levels of CM were more likely to display greater levels of aggression and were also characterized by lower protective clinical scores on risk assessment. Surprisingly, CM did not play a significant role in changes in risk and protective factors over the treatment trajectory.

When investigating the association between CM and self-reported inpatient aggression in the VRAPT sample, we found that higher levels of CM were associated with higher scores on general aggression, which is consistent with previous findings [e.g., (80)]. Furthermore, we found that higher levels of CM were associated with both subtypes of aggression, namely reactive and proactive aggression. This finding is not entirely in line with the findings reported in the literature. More precisely, consistent with previous findings [e.g., (81)], higher levels of CM were associated with higher levels of reactive aggression. However, we also found a significant positive association between CM and proactive aggression, which was not documented in previous research. This might be explained by the type of patients in our sample, who have frequently experienced violence in their history and still experience it in the present. Previous research that only showed a link with reactive aggression was mainly conducted among the general population or psychiatric patients in regular mental health care. It could be that forensic psychiatric patients are more prone to exhibit both proactive aggression and reactive aggression due to their diverse psychiatric diagnoses and a history of violent behavior (every patient in an FPC has a history of violent crime). In addition, this may be more pronounced in patients who have experienced more ACEs because of the disruptions ACEs can cause in regulating systems. Also, the mediating factor of the Hostile Attribution Bias, which is well documented to be prevalent in forensic psychiatric samples and higher in those who experienced ACE (81, 82), might impact the choice to proactively display aggression.

In our study, we found positive correlations between the total CM score and its five subtypes, with general aggressive behavior. Physical neglect had the strongest correlation, followed by physical abuse, emotional abuse, emotional neglect, and physical neglect. Most CM categories were significantly and positively correlated with reactive and proactive aggression, except for sexual abuse, which did not correlate with both types of aggression. Emotional neglect was solely correlated with proactive aggression. Nevertheless, our findings align considerably with earlier research showing that CM is associated with problems in aggression regulation later in life (15). This aggression dysregulation in patients who experienced more childhood adversity may indicate DTD, reflecting dysregulation in internal and psychological systems (nervous system, stress system, emotion regulation) due to early trauma and neglect (3, 11).

Unexpectedly, the total CM score was not associated with staff-reported inpatient aggression scores. However, some CM subcategories did correlate with both the staff-reported weekly peak score and the general weekly aggression score. This was the case for physical neglect and emotional abuse. Physical abuse was correlated with the staff-reported general week scores, but not with the peak scores. Sexual abuse and emotional neglect were not associated with staff-reported inpatient aggression. Interestingly, these two subcategories were not correlated with self-reported reactive aggression either, or with self-reported proactive aggression in the case of sexual abuse. Previous research has linked both categories to aggression in later life (83, 84). It remains unclear why this finding was not replicated in our forensic group. Forensic patients with a history of sexual abuse are more likely to commit sexual crimes in later life [e.g., (85)], which might be a different type of aggression than assessed with the questionnaires on aggression used in our study.

Besides these links to aggression, we also investigated how CM is linked to risk and protective factors for recidivism within the HKT-R sample. The results showed a clear connection between higher reported CM and an elevated likelihood of criminal recidivism. Our study reinforces previous findings signifying the role of ACEs in amplifying recidivism risk [e.g., (52)], warranting their recognition as important targets for intervention in offender rehabilitation programs. The association of CM with both risk and protective factors emphasizes the importance of considering these factors when providing forensic treatment for traumatized offenders. However, in this study, we did not investigate a direct link between CM and the likelihood of recidivism. Consequently, no conclusion can be drawn regarding whether CM should be targeted as a risk factor or, perhaps, be better considered as a responsivity factor. This is important to address in future research. Furthermore, in general, both samples displayed a notable prevalence of CM, aligning with prior studies in forensic populations [e.g., (13, 14)], while surpassing ACEs prevalence in the general population (86). Emotional neglect emerged as the most prevalent form of CM in both samples (see Supplementary Tables S2–S4 for more detail), a contrast to studies in prison populations, which typically emphasize higher abuse than neglect prevalence (87, 88). This might suggest that forensic psychiatric patients encounter childhood neglect more frequently than “regular” prisoners. Furthermore, considering risk and protective subscales separately, only the protective factors had a significant negative correlation with CM. This means that patients with higher levels of CM have less protection against reoffending after release from forensic psychiatric institutions. The correlation between CM and risk factors was not significant, implying that CM predominantly impacts the development of positive life skills that protect the patient against poor life decisions like criminality, without necessarily exacerbating existing risk factors.

Surprisingly, none of the CM subcategories were associated with the general risk of reoffending, or risk and protective factors. This indicates that experiencing one type of childhood maltreatment may not necessarily increase the risk of reoffending, but a combination of different types of CM could contribute to the occurrence of (re)offending behavior.

As the most prevalent type of CM was emotional neglect, one could imagine that emotional neglect—especially in combination with other ACEs—has a negative impact on developing positive life skills. Neglect is known to be one of the most impactful childhood experiences, impacting the development of empathy, morality, and social connectedness or attachment as relevant factors to forensic psychiatry [e.g., (89, 90)]. Children who have been neglected do not have (enough) adult guidance in developing these important characteristics, all connected to delinquency and antisociality. Research also shows that childhood trauma and abuse are associated with these characteristics [e.g., (46, 91)]. As previously mentioned, the dysregulation caused by not being raised in a safe, consistent and connected environment might result in the development of characteristics that make patients more likely to commit a crime and reoffend. Therefore, it is important to (if possible, preventively) target these resulting characteristics and symptoms developed by experiencing childhood adversity. However, in our study, only 4% of the HKT sample and 7% of the VRAPT sample were diagnosed with PTSD. This is remarkable, considering the high level of CM (83% and 65%) in both samples and its impact on the level of aggression and criminal risk factors. This underlines the importance of taking trauma and neglect into account in the (forensic) diagnostic process and looking beyond the narrow classification of PTSD and symptoms when assessing and treating (forensic) psychiatric patients. ACEs might not lead to PTSD as we classify them, but they are linked to more problems and higher risk in forensic patients.

It is possible that symptoms and problems that arise from ACEs are classified as personality disorders, the most diagnosed disorders in our samples. It is important to consider that this personality dysfunction might be influenced by the disturbances in functioning caused by experiencing childhood adversity. Several of the criminogenic factors and personality disorder characteristics correspond with symptoms listed in complex PTSD and the proposed DTD classification (11). They could be a result of the dysregulation in several neurobiological and psychological systems known to be associated with childhood adversity (92, 93). It is important to note that the ramifications of ACEs have not been comprehensively integrated into the DSM-5 (94) and treatment guidelines. To address this gap, an encompassing classification termed DTD has been proposed for potential inclusion in the DSM-5 (11). Although DTD is still not included in the DSM-5, contemporary studies have proliferated to substantiate DTD classification [e.g., (95)]. This progress led the ICD-11 to introduce a classification termed “complex PTSD” (96) including similar symptoms to DTD. While PTSD-targeted treatment is empirically grounded and beneficial, it may be inadequate for individuals with pervasive ACEs. “Trauma-informed” therapy and interventions have exhibited promise in providing comprehensive care to help these individuals in transcending the consequences of childhood adversity and fostering healthier lives (97).

Finally, considering treatment trajectories, we found that CM did not influence the changes over time in clinical risk factors overall or risk and protective factors separately. Both groups (CM and no CM) had a similar trajectory, in which the HKT-R clinical scale and risk subscales decreased whereas the protective scale increased throughout treatment. In general, most of the progress was achieved between admission (T1) and unguided leave (T2). After this period, the progress was less obvious. These findings are consistent with previous research [e.g., (44, 47)].

However, when looking at the length of institutional stay, we found that this was significantly longer for the CM group than the no CM group. This was expected on the basis of previous research in general mental health care finding similar results [e.g., (59)]. Especially the duration between the start of unguided leave (T2) and finishing the tbs. trajectory at unconditional discharge (T3) was longer for the CM group, although this is the period in which—as mentioned above—lesser change takes place when measured by risk (and protective) factors. It is interesting to consider why this part of the treatment trajectory takes longer for patients who have experienced CM. As the patients take more unguided resocialization steps into society from this point on, it could be that this group struggles more when being on their own again, which can be tied to the importance of social support especially for people with ACEs (98). Another possibility is that they still need more guidance and therapy during this period than the no CM group, because of their complex profiles, lengthening the trajectory. The intensity of the treatment and the types of interventions and guidance provided were not known to the authors, so we cannot base any conclusions about this on our data.

### 4.2 Clinical implications

Our findings underline the importance of a careful assessment of patients in forensic care, taking into account personal histories. Many of them have experienced hardship, unsafety and other forms of neglect and abuse while growing up. This impacts their global functioning and influences the offense chain, ergo their path toward delinquency and the index offense for which they receive treatment. Their self-reports of abuse and neglect show higher rates of childhood adversity than when scored by professionals, indicating that there is still work to be done to get a correct and full picture of these patients.

The impact of ACEs on aggression and criminogenic (risk and protective) factors should be taken into account when treating forensic patients, as it might be used to help them and their therapists understand their problems better and more in depth. Therapies aimed at personality disorders, like (forensic) schema therapy, are already used in forensic practice and target ACEs in therapeutic interventions like imaginary rescripting, also when no PTSD is diagnosed. Our results underline the importance of these types of therapy.

As it has developed in general psychological and psychiatric care in recent decades, trauma-informed care should also be part of forensic psychiatric treatment. Because childhood adversity does not seem to influence treatment trajectory course in patients successfully completing the tbs. trajectory, although it does influence the institutional length of stay, it is possible that the current forensic psychiatric system already has a good basis for this that can be further developed.

### 4.3 Limitations and directions for future research

An important limitation of this study is that we investigated treatment trajectories only in the sample of patients who had successfully progressed in their treatment and were unconditionally released from the FPCs (The HKT-R sample). The risk assessment scores of patients still residing in FPCs (like those from the VRAPT sample) were unfortunately not available to the researchers. Also, no information was available on patients residing in long care or long stay facilities. These patients are all still at high risk of reoffending and some of them could stay in highly secured residential care for the rest of their lives. They never reach unconditional discharge (T3). It is possible that these patients have higher ACE and CM scores, or that their continuing higher risk and lower protective factor scores lead to different results concerning the link between ACEs and treatment progress compared to unconditionally released patients.

Our datasets included, as described, only information on childhood maltreatment, not on household dysfunction. Usually both broad categories of ACE are taken into account when researching the impact of ACE. Our study was therefore somewhat limited to CM only when looked at from the broader ACE perspective.

The study’s scope was constrained by its reliance on correlational analyses for investigating associations between pairs of variables. As a result, potential confounding variables such as psychiatric diagnosis, gender, and education were not considered. For example, in the VRAPT sample, completed lower education was more common than in the HKR-R sample, indicating that the level of education can vary greatly even among forensic patients themselves. In addition, ACEs have demonstrated significant associations with depression, bipolar disorder, suicide, substance misuse (99–101), and borderline personality disorder (102), with higher prevalence among females (103) and less educated individuals (104). Likewise, due to the correlational design of our study, causal inferences cannot be drawn. However, the study’s merit lies in establishing correlations between focal variables, a crucial step preceding linear regression and more complex models.

Another limitation is that both datasets have missing values, which resulted in smaller than expected samples for some of the analyses (though still large enough). Furthermore, the HKT-R was assessed retrospectively by trained coders, however, we did not calculate inter-rater reliability in this sample. In a comparable sample of forensic patients finishing their treatment trajectory between 2004 and 2008, the inter-rater reliability was very good for all items used (62). Since the training and coding procedures were based on the same format in both studies, we could only assume good inter-rater reliability for the currently used 2009–2013 dataset.

Since the LGCM for clinical and protective scales did not fit the data well, we were forced to opt for a simpler model of a repeated measures ANOVA within- and between-subjects design to investigate treatment trajectories. To do this, we dichotomized the ACE variable. As the incidence and severity of childhood adversity were very high in both samples, it is possible that the remaining sample of patients with no to minimal childhood adversity (78 patients, only 17% of the sample) was too small to have sufficient power for analyses. This might explain non-significant results or small effect sizes, especially in the analyses on treatment trajectories, where a dichotomous grouping variable was used for ACE instead of the continuous variable based on severity. In this study, treatment progress was only defined by a decrease in risk factors and an increase in protective factors. This might be a rather limited view of what constitutes treatment progress. As mentioned in the discussion on the treatment duration, it would be interesting to see how the ACE and no ACE groups – or better, a continuous scale of ACE scores—differ in the types and intensity of therapy and guidance provided within the tbs. trajectory. It would also be interesting to apply the LGCM to different types of ACEs besides the compound factor we used in this first study on this subject. Lastly, our patient samples come from a forensic psychiatric system (tbs) that is only known in the Netherlands. It is uncertain how these results generalize across different groups of forensic psychiatric patients, offenders and inmates.

The discussed limitations outline several avenues for future research. For example, it would be interesting to study treatment trajectories and potential mitigation of (all) ACEs in a (large) sample of the entire population of forensic psychiatric patients, and not just those who have completed the treatment trajectory. It would also be important to investigate the influence of all ACEs, including both household dysfunction as well as both childhood abuse and childhood neglect separately on later well-being, problematic characteristics and changes in risk and protective factors. The predictive value of ACEs on aggression and forensic risk factors could be looked into, adding to the associations we found in this first study.

Criminal risk factors are mostly behavioral factors. As mentioned above, there is more to treatment progress than only improvement in behavioral measures. We would advise future researchers to focus on several markers of treatment progress, like patient well-being and level of psychiatric problems. Childhood adversity has an impact on the brain, the nervous system and physical functioning. Recent research has focused on the association between ACEs, disturbances in physical systems and aggression and personality disorders in general and in forensic patients (43, 92). Besides behavioral measures, physiological biomarkers like heart rhythm, skin conductance and neurological functioning should be included in more research to provide greater insight into complex associations between ACEs and risk behaviors like delinquency.

Finally, it would be beneficial to investigate what works as a “buffer” for the impact of ACEs on early and later life. This has only been the subject of research in recent years [e.g., (89, 105)]. For instance, Crouch et al. (106) found that safe, stable, and nurturing relationships can mitigate adverse physical and mental health outcomes following ACEs exposure. Similarly, Hughes et al. (107) noted that experiencing four or more ACEs correlated with negative psychological impacts, yet consistent involvement in sports substantially reduced this association. Not all children and adolescents encountering ACEs manifest unfavorable outcomes in later life. Therefore, further research is imperative to uncover factors fostering resilience and potentially moderating the long-term impact of ACEs. Such insights could facilitate the development of enhanced preventive and therapeutic strategies.

### 4.4 Conclusion

Severe childhood abuse and (especially emotional) neglect were highly prevalent in the samples of forensic psychiatric patients we studied. We found that patients who experienced more CM, reported and displayed more aggression. Their scores on criminal risk assessment were higher compared to patients with less or no CM. Patients with higher levels of CM developed less positive life skills to build protective factors against criminality and recidivism. The presence of CM did not influence the forensic treatment trajectory for those who completed treatment, but it did influence institutional length of stay, which was longer for the CM group. Our findings could be relevant for forensic clinical practice, as they show the importance of histories of childhood adversity in forensic psychiatric patients and the relevance of ACEs—possibly also without PTSD diagnosis—for forensic psychiatric treatment settings. However, the present study has some limitations and before drawing any firm conclusions, further research is needed to replicate these results. This can ultimately lead to the incorporation of useful corresponding practices in forensic psychiatric treatment.

## Data availability statement

The original contributions presented in the study are included in the article/Supplementary material, further inquiries can be directed to the corresponding author.

## Ethics statement

The studies involving humans were approved by the Medical Ethical Committee of the University Medical Center Groningen, the ethical review board of Tilburg University, the Ministry of Justice and Security and the scientific research committee of Fivoor. The studies were conducted in accordance with the local legislation and institutional requirements. The participants provided their written informed consent to participate in this study.

## Author contributions

MK and SB designed the study and formulated research questions and hypotheses. MK and MJ analyzed the data. MK wrote the first draft of the manuscript and updated each following version after critical revision from MJ and SB and independent reviewers. All authors contributed to the article and approved the submitted version.

## Footnotes

^1^Demographics of the VRAPT dataset were not available for the researchers in detail and were therefore partly derived from the primary research article about VRAPT: Klein (57).
