# Supplementary material for: The impact of childhood maltreatment on aggression, criminal risk factors, and treatment trajectories in forensic psychiatric patients
Source: Front Psychiatry. 2023 Nov 30;14:1128020. doi: 10.3389/fpsyt.2023.1128020 (PMC10720334; doi:10.3389/fpsyt.2023.1128020)
Supplement: Supplementary file 1 [file Data_Sheet_1.docx]

Supplementary Material

The Impact of Childhood Maltreatment on Aggression, Criminal Risk Factors and Treatment Trajectories in Forensic Psychiatric Patients

**Marijtje Koolschijn, Marija Janković, Stefan Bogaerts***

*** Correspondence:** [S.Bogaerts@tilburguniversity.edu](mailto:S.Bogaerts@tilburguniversity.edu)

# Supplementary Tables

Table S1

Values of Skewness and Kurtosis for All Study Variables

|  | Skewness | Kurtosis |
| --- | --- | --- |
| HKT-R dataset |  |  |
| CM (H07) total score | 0.00 | -1.11 |
| Emotional abuse | 1.433 | 0.054 |
| Physical abuse | 0.348 | -1.887 |
| Sexual abuse | 1.326 | -0.243 |
| Emotional neglect | -0.339 | -1.893 |
| Physical neglect | 2.231 | 2.992 |
|  |  |  |
| Total clinical scale |  |  |
| T1 | 0.68 | -0.26 |
| T2 | 0.89 | 0.52 |
| T3 | 1.63 | 3.02 |
| Risk subscale |  |  |
| T1 | -0.09 | -0.31 |
| T2 | -0.26 | -0.24 |
| T3 | -0.61 | 0.00 |
| Protective subscale |  |  |
| T1 | 0.51 | -0.08 |
| T2 | 0.52 | 0.23 |
| T3 | 1.14 | 1.38 |
|  | Skewness | Kurtosis |
| VRAPT dataset |  |  |
| RPQ total score | 0.100 | -0.249 |
| RPQ reactive aggression | -0.410 | -0.262 |
| RPQ proactive aggression | 0.454 | -0.534 |
|  |  |  |
| CTQ total score | 1.117 | 1.016 |
| Emotional abuse | -0.068 | -1.698 |
| Physical abuse | 0.347 | -1.736 |
| Sexual abuse | 1.269 | -0.119 |
| Emotional neglect | -0.712 | -1.089 |
| Physical neglect | 0.201 | -1.460 |
|  |  |  |
| SDAS peak score | 0.894 | 0.485 |
| SDAS average score | 1.071 | 0.569 |

*Note.* T1 = admission to clinic; T2 = unguided leave; T3 = unconditional discharge. CM as scored on H07 – childhood victimization. RPQ = Reactive Proactive Questionnaire. CTQ = Child Trauma Questionnaire, Short Form. SDAS = Social Dysfunction and Aggression Scale.

Table S2

Prevalence and severity of CM, as reported on the CTQ-SF (VRAPT sample)

| Prevalence and severity  n (%) | Total score  (n=103) | Emotional abuse  (n=110) | Physical abuse  (n=108) | Sexual abuse  (n=110) | Emotional neglect  (n=109) | Physical neglect  (n=109) |
| --- | --- | --- | --- | --- | --- | --- |
| None to minimal | 41 (35%) | 39 (36%) | 54 (50%) | 76 (69%) | 21 (19%) | 41 (38%) |
| Slight to moderate | 20 (17%) | 12 (11%) | 9 (8%) | 9 (8%) | 13 (12%) | 18 (17%) |
| Moderate to severe | 27 (23%) | 22 (20%) | 10 (9%) | 7 (6%) | 20 (18%) | 29 (27%) |
| Severe to extreme | 15 (13%) | 37 (34%) | 35 (32%) | 18 (16%) | 55 (51%) | 21 (19%) |

*Note.* CTQ-SF = Child Trauma Questionnaire – Short Form.

Table S3

Prevalence and severity of CM in percentages, as scored on the HKT-R (HKT-R sample)

| Prevalence and severity  n (%) | Total score  (n=468) |
| --- | --- |
| Never (0) | 78 (17%) |
| Incidental (1) | 70 (15%) |
| Chronic neglect (2) | 152 (32%) |
| Chronic abuse (3) | 57 (12%) |
| Chronic neglect and abuse (4) | 111 (24%) |

*Note.* HKT-R = Historische Klinische Toekomst – Revisie (Historical Clinical Future – Revision).

Table S4

Prevalence of CM subcategories in percentages, as scored on the HKT-R (HKT-R sample)

| Prevalence  n (%) | Emotional abuse  (n=468) | Physical abuse  (n=468) | Sexual abuse  (n=468) | Emotional neglect  (n=468) | Physical neglect  (n=468) |
| --- | --- | --- | --- | --- | --- |
| Yes | 370 (79%) | 274 (58%) | 363 (78%) | 195 (42%) | 408 (87%) |
| No | 98 (21%) | 194 (42%) | 105 (22%) | 273 (58%) | 60 (13%) |

*Note.* HKT-R = Historische Klinische Toekomst – Revisie (Historical Clinical Future – Revision).

Table S5

Psychiatric Diagnoses in the VRAPT Sample

| Psychiatric diagnosis | *N* (%) |
| --- | --- |
| Clinical disorders |  |
| Schizophrenia | 26 (20.3 %) |
| Schizoaffective disorder | 2 (1.6 %) |
| Delusional disorder | 2 (1.6 %) |
| Psychotic disorder not otherwise specified | 10 (7.8 %) |
| Autism spectrum disorder | 17 (13.3 %) |
| Attention-deficit/hyperactivity disorder | 19 (14.8 %) |
| Pedophilia | 8 (6.3 %) |
| Other paraphilias | 5 (3.9 %) |
| Personality disorders |  |
| Paranoid personality disorder | 1 (0.8 %) |
| Antisocial personality disorder | 48 (37.5 %) |
| Borderline personality disorder | 16 (12.5 %) |
| Narcissistic personality disorder | 7 (5.5 %) |
| Dependent personality disorder | 2 (1.6 %) |
| Personality disorder not otherwise specified | 52 (40.6 %) |

*Note*. As there is overlap in classifications, not all percentages add up to 100%.

Table S6

Psychiatric Diagnoses in the HKT-R Sample

| Psychiatric Diagnoses | *N* (%) |
| --- | --- |
| Axis I diagnosis |  |
| Developmental disorder | 43 (9.2 %) |
| Substance use disorder | 220 (47 %) |
| Psychotic disorder | 124 (26.5 %) |
| Mood disorder | 44 (9.4 %) |
| Other Axis I disorders | 67 (14.3 %) |
| Axis II diagnosis |  |
| No diagnosis |  |
| Cluster A personality disorder | 16 (3.4 %) |
| Cluster B personality disorder | 143 (30.6 %) |
| Cluster C personality disorder | 16 (3.4 %) |
| Personality disorder not otherwise specified | 162 (34.6 %) |
| Multiple personality disorders | 13 (2.8 %) |
| Intellectual disability | 72 (15.4 %) |

*Note*. As there is overlap in classifications, not all percentages add up to 100%.

# Latent Growth Curve Analysis

## Unconditional Models

First, we used LGCM to test an unconditional model with a simple linear trajectory of the total clinical scale score for the entire HKT-R sample at the three time points, namely admission, unguided leave, and unconditional leave (Figure 1). However, this hypothetical model did not correspond well with the empirical data due to insufficient fit values (CFI = .83, SRMR = .07). Therefore, we opted for a simpler form of a repeated measures design, the analysis of variance (ANOVA) on repeated measures (within-subject). In addition to the assumption tests already conducted, normality checks were performed on the residuals, which were approximately normally distributed.

Mauchly's test of sphericity was statistically significant (p < .05), meaning that the assumption of sphericity was violated. To reduce the risk of type I error (false-positive conclusion), we used the Huynh-Feldt correction as this is recommended when epsilon is > 0.75 (Girden, 1992). The ANOVA showed that the mean score on the clinical scale decreased significantly at the three time points [F(1.863, 428.600) = 149.088, p < 0.001, η2 = .393]. To substantiate this, the Bonferroni correction was applied as post-hoc test. This showed that the clinical scale score decreased significantly (p < .05) from T1 to T2 by an average of 6.463 and further decreased from T2 to T3 by an additional 3.483.

Figure S1

Growth Trajectory of the Clinical Scale

*Note*. CM = Childhood maltreatment; T1 = Admission to the clinic; T2 = Unguided leave; T3 = Unconditional discharge.

To research whether the data could be modeled in a better way by doing so, we looked at subscales comprised of the risk and protective items separated. This has been done in previous research as mentioned before, see Bogaerts et al., 2020.

We examined an unconditional model with a simple linear trajectory of the risk subscale score at three time points (Figure 2), which resulted in an acceptable model fit (CFI = .90, SRMR = .05). The risk subscale score decreased significantly (*p* < .001) from admission to the clinic until unconditional release, with approximately 2.03 points at each time point, *b* = - 2.03, *p* < .001. The variance around the slope factor was not statistically significant (*b* = 1.33, *SE* = 1.18, *p* = .15), meaning that all patients have a similar progress/growth rate.

Figure 2

Growth Trajectory of the Risk Subscale

*Note*. CM = Childhood maltreatment; T1 = Admission to the clinic; T2 = Unguided leave; T3 = Unconditional discharge.

Lastly, a simple linear trajectory of the protective subscale score at three time points was tested (Figure 3). The model did not have good fit to the data (CFI = .74, SRMR = .08). Therefore, we conducted ANOVA on repeated measures (within-subject). Mauchly's test of sphericity was statistically significant (p < .05), meaning that the assumption of sphericity was violated. Because epsilon was larger than 0.75, we used Huynh-Feldt correction. In addition, Bonferroni correction was applied for post-hoc test. The results showed the protective subscale increased significantly from T to T3[F(1.784, 162.385) = 68.437, p < 0.001, η2 = .429]. Post-hoc analysis revealed that the protective subscale increased significantly from T1 to T2 by an average of 3.995 and further increased from T2 to T3 by an additional 2.033.

Figure 3

Growth Trajectory of the Protective Subscale

*Note*. CM = Childhood maltreatment; T1 = Admission to the clinic; T2 = Unguided leave; T3 = Unconditional discharge.
